# Does co-transcriptional regulation of alternative splicing mediate plant stress responses?

**DOI:** 10.1093/nar/gkz121

**Published:** 2019-02-22

**Authors:** Ibtissam Jabre, Anireddy S N Reddy, Maria Kalyna, Saurabh Chaudhary, Waqas Khokhar, Lee J Byrne, Cornelia M Wilson, Naeem H Syed

**Affiliations:** 1 School of Human and Life Sciences, Canterbury Christ Church University, Canterbury, CT1 1QU, UK; 2 Department of Biology and Program in Cell and Molecular Biology, Colorado State University, Fort Collins, CO 80523-1878, USA; 3 Department of Applied Genetics and Cell Biology, University of Natural Resources and Life Sciences - BOKU, Muthgasse 18, 1190 Vienna, Austria

## Abstract

Plants display exquisite control over gene expression to elicit appropriate responses under normal and stress conditions. Alternative splicing (AS) of pre-mRNAs, a process that generates two or more transcripts from multi-exon genes, adds another layer of regulation to fine-tune condition-specific gene expression in animals and plants. However, exactly how plants control splice isoform ratios and the timing of this regulation in response to environmental signals remains elusive. In mammals, recent evidence indicate that epigenetic and epitranscriptome changes, such as DNA methylation, chromatin modifications and RNA methylation, regulate RNA polymerase II processivity, co-transcriptional splicing, and stability and translation efficiency of splice isoforms. In plants, the role of epigenetic modifications in regulating transcription rate and mRNA abundance under stress is beginning to emerge. However, the mechanisms by which epigenetic and epitranscriptomic modifications regulate AS and translation efficiency require further research. Dynamic changes in the chromatin landscape in response to stress may provide a scaffold around which gene expression, AS and translation are orchestrated. Finally, we discuss CRISPR/Cas-based strategies for engineering chromatin architecture to manipulate AS patterns (or splice isoforms levels) to obtain insight into the epigenetic regulation of AS.

## INTRODUCTION

Alternative splicing (AS) is an important gene regulatory process that generates multiple transcripts from a single gene (1–5). The constitutive splicing process uses only one set of splice sites to generate a single mRNA, whereas AS uses different combinations of splice sites to produce a few to hundreds of mRNA isoforms from one gene (3). AS is a widespread mechanism in higher eukaryotes, regulating up to 95% of human and 70% of plant multi-exon genes (2,6–9). Several studies suggest that plants use AS to fine-tune their physiology and metabolism, thereby maintaining a balance between carbon fixation and resource allocation under normal and stress conditions including cold, drought, heat, high salinity and pathogen infection (1,3,10–17). Further interest in AS has been rekindled with the discovery that temperature-dependent AS plays an important role in regulating transcript levels of key circadian clock genes in plants (15,16,18,19). However, the molecular mechanisms by which AS regulates these responses are poorly understood. Intriguingly, the majority of genes encoding splicing regulators in plants are also subject to extensive AS, and their splicing patterns are altered in response to various environmental stresses (20–22).

In metazoans, the splicing process is predominantly co-transcriptional (23–26). Collective data from mammalian studies on chromatin structure, histone modifications and transcription elongation rate point toward epigenetic control as a key component of AS regulation in a cell- and condition-dependent manner (27–31). Additionally, the link between RNA modifications (the epitranscriptome) and the transcription machinery may have a strong bearing on splicing and translational regulation (32,33). This is intriguing because dynamic crosstalk between transcription, splicing and translation is likely to confer an additional advantage, and only transcripts with appropriate modifications in a given condition may be processed and/or translated. As sessile photosynthetic organisms, plants likely exploit this dynamic crosstalk to fine-tune their metabolism and physiology for rapid adaptation to changing environments. Indeed, evidence in support of crosstalk at the co/post-transcriptional level through epigenetic modifications and splicing are beginning to emerge in plants (34–36). However, how stress modulates the underlying regulatory networks and crosstalk with global AS profiles needs further research. Although variation in DNA sequence can influence the splicing outcome, we postulate that generation of AS variation via chromatin modifications rather than nucleotide sequence variation provides plants with flexibility in reprogramming gene expression to ensure appropriate responses to changing growth conditions. Recent evidence also show that plants exhibit dynamic DNA methylation and epigenetic modifications under different conditions (34,37–41). Since differential DNA methylation patterns and histone modifications are strongly correlated with nucleosome occupancy (42), they may influence RNA polymerase II (pol II) elongation speed and splicing factor recruitment, resulting in different splicing outcomes. Therefore, co-transcriptional splicing and its modulation by different epigenetic and epitranscriptomic modifications in response to diverse environmental cues may be a preferred mechanism to achieve optimal gene expression levels in plants. Furthermore, condition-dependent epigenetic changes may also help plants to remember past stresses (i.e. stress memory) (40,43–45) and rapidly employ appropriate transcriptome responses to subsequent stresses. In this review, we discuss the current status of chromatin-mediated regulation of co/post-transcriptional processes, with emphasis on how crosstalk between various epigenetic, epitranscriptomic modifications, and the splicing machinery modulates transcript diversity, abundance and stability.

## OVERVIEW OF PRE-mRNA SPLICING

Pre-mRNA splicing is catalyzed by the spliceosome, a large ribonucleoprotein complex that recognizes various *cis*-sequences in pre-mRNAs, including 5′ and 3′ splice sites, branch points, polypyrimidine tracts and other splicing regulatory elements (suppressors and enhancers) (46–52). The core spliceosome is composed of five uridine-rich small nuclear ribonucleoproteins (snRNPs U1, U2, U4, U5 and U6) and additional spliceosome-associated proteins (53,54). Other non-snRNP splicing factors (SFs), predominantly serine/arginine-rich (SR) proteins and heterogeneous nuclear ribonucleoproteins (hnRNPs), target splicing enhancers and suppressors located in exons and introns, and regulate splice site selection by the spliceosome (52,53,55).

AS occurs when the spliceosome differentially selects splice sites. Common types of AS include exon skipping (ES), mutually exclusive exons (MXE), intron retention (IR), and selection of alternative donor (Alt5′) and acceptor splice (Alt3′) sites (56). Recently characterized exitrons (EIs) complement the repertoire of AS events (57,58). EIs are alternatively spliced internal regions of reference protein-coding exons. Majority of EIs have lengths divisible by three and they broadly impact protein function by affecting protein domains, disordered regions and the availability of sites for various post-translational modification (PTM) (57).

Different splice isoforms display various fates in plants that may include (i) nuclear sequestration and further splicing to generate full-length mRNAs (59,60), (ii) translation into functional or truncated proteins (10,61,62) and (iii) degradation via nonsense-mediated mRNA decay (NMD) (63–69). Regulation of AS and the fate of alternatively spliced transcripts are mainly driven by the concentration of SFs and their proportions (largely due to competition between SR proteins as positive regulators and hnRNPs as negative regulators for binding to *cis*-regulatory elements) in particular cell types/conditions. Additionally, the structure of pre-mRNAs also regulates splicing significantly (70,71). In both mammals and plants, chromatin, which carries differential DNA methylation and multiple histone modifications, may mediate pol II processivity to influence splicing outcomes (35,72–79). Hence, splicing regulation is mediated through a complex cellular network referred to as the ‘splicing code’ that fine-tunes gene expression in response to different conditions (80,81).

## CO-TRANSCRIPTIONAL REGULATION OF ALTERNATIVE SPLICING

An extensive body of evidence suggests that splicing is predominantly coupled to transcription in metazoans, and is dependent on chromatin structure, which is modulated by DNA methylation, histone PTM and chromatin adapter complexes (48,82–85). The C-terminal domain (CTD) of pol II serves as a landing pad for the recruitment of proteins involved in capping, splicing, polyadenylation and export of transcripts (75,86,87). Various studies have shown that pol II CTD phosphorylation facilitates the recruitment of SFs including SR proteins to influence both constitutive and alternative splicing (88–91). Recruitment and kinetic models have been proposed to explain the mechanism by which transcriptional machinery controls AS (27,31,92,93). The recruitment model states that the transcription machinery interacts directly or indirectly with SFs and thereby affects splicing outcomes. The kinetic model proposes that decreasing the speed of pol II allows additional time for an upstream exon with weak splice sites to recruit the splicing machinery before a downstream exon with stronger splice sites emerges during pre-mRNA synthesis (94,95).

Similar to mammals (96), very recent native elongating transcript sequencing (NET-seq) data from Arabidopsis also showed that phosphorylation of pol II at serine 5 (Ser 5P) mediates interactions with the spliceosome (97). In addition, pol II elongation speed in Arabidopsis was also found to be slower in exons than introns, facilitating exon and splice site recognition. Accumulation of pol II Ser 5P at 5′ splice sites, in concert with the splicing machinery, facilitates 5′ splice site recognition and cleavage during elongation (97). Interestingly, plants can employ a signaling molecule from chloroplasts to regulate AS in the nucleus under different light conditions (13). The nature of this chloroplast-derived retrograde signal is not clear, although a nuclear regulatory mechanism that affects AS of a subset of Arabidopsis genes has been revealed (13,98). Interestingly, pol II elongation speed is faster under light conditions than in darkness. In addition, greater pol II processivity is associated with a more open chromatin structure, which favors pol II elongation (13,98). These results provide strong evidence that plants can control nuclear events such as AS by coupling environmental and physiological cues to pol II elongation speed, and thereby elicit an appropriate plant responses (13,98–100). Similarly, the spliceosome disassembly factor NTR1 is essential for appropriate expression and splicing of the *DELAY OF GERMINATION 1* (*DOG1*) gene. AtNTR1-deficient plants display a higher pol II elongation rate, preference for downstream 5′ and 3′ splice sites, and increased exon skipping (101). Interestingly, AtNTR1 also co-localizes with pol II to achieve splicing of target genes (101). Recent data from plants have also identified a strong relationship between chromatin changes, transcriptional control and AS regulation. For example, quantitative variation in the transcription of the *FLOWERING LOCUS C* (*FLC*) gene in Arabidopsis was associated with H3K36me3 and H3K4me2 histone marks, suggesting that different chromatin states influence initiation and elongation rates that affect splicing of *FLC* (102). Chromatin-bound RNA was more abundant inside exon 1 of *FLC* than at the exon1–intron1 junction, suggesting that splicing at intron 1 is mostly co-transcriptional (102). Additionally, *FLC* intron 1 retention is associated with a high level of H3K27me3, which is coincident with low cytosine-guanine(CG) methylation and H3K36me3/H3K4me1 marks, demonstrating a link between chromatin features and splicing outcomes in the *FLC* gene (103). Recently, Ullah *et al.* (35) investigated the relationship between open chromatin and intron retention in Arabidopsis and rice. They showed that the chromatin structure is more open in retained introns. Based on this correlation, it was suggested that the open chromatin architecture in retained introns enhances the pol II elongation rate, which leads to skipping of splice sites by the spliceosome (35). Together these studies strongly suggest that splicing is also co-transcriptional in plants, and that the chromatin environment has a strong effect on pol II processivity to modulate the transcriptional and splicing dynamics of plant genes.

## DNA METHYLATION AND REGULATION OF ALTERNATIVE SPLICING

Plants exhibit extensive variation in DNA methylation and gene expression under different developmental and stress conditions (104–108). In eukaryotes, DNA methylation occurs in symmetric CG and CHG (H = A, T or C) and asymmetric CHH contexts (109). However, DNA methylation is largely dependent on the CpG context in plants. In the Arabidopsis genome, 24% of CG sites are methylated, compared with only 6.7% of CHG and 1.7% of CHH sites (110,111). Interestingly, nucleosomal DNA is highly methylated, and exons rather than the introns are marked at the DNA level by high occupancy of nucleosomes. These are preferentially positioned at intron–exon and exon–intron boundaries in both mammals and Arabidopsis (42,77,112,113). Additionally, nucleosome occupancy is also lower in alternatively spliced exons compared with constitutively spliced exons (77–79,114,115). Since DNA is packaged into nucleosomes, pol II elongation rate is inherently subject to frequent pausing at constitutively spliced exons with high GC levels (116,117), and regions of high nucleosome density slow down pol II to facilitate the recruitment of SFs to weaker upstream splice sites (24,28,79,114).

An example of this is found in the honeybee, in which DNA methylation is almost exclusively found in exons with a strong correlation between methylation patterns on alternative exons and splicing patterns of these exons in workers and queens (73). Intriguingly, a reduction in methylation of the *dnmt3* gene encoding a methyltransferase via RNAi results in widespread changes in AS in honeybee fat tissues (118). Additionally, a DNA-binding protein, CCCTC-binding factor (CTCF), promotes inclusion of weak upstream exons in the *CD45* gene by causing local pol II pausing in mammals. Methylation of exon 5 abolished CTCF binding and resulted in the complete loss of exon 5 from *CD45* transcripts (28). Interestingly, a direct link was very recently unveiled between DNA methylation and AS in humans by perturbing DNA methylation patterns of alternatively spliced exons. In this study, the authors used CRISPR-dCas9 proteins (for details, see the ‘Engineering splicing variation’ section below) and methylating/demethylating enzyme fusions (119). This work clearly demonstrates that changes in the methylation pattern of alternatively spliced exons mediates their inclusion, but has no effect on introns or constitutively spliced exons (119).

Recent work in plants demonstrated abundant DNA methylation and splicing variation under different growth and stress conditions, and during different developmental stages. For example, quantification of AS in wild-type (WT) and *OsMet1-2* (CG methyltransferase mutant) rice lines revealed widespread differences in splicing variation (120). Consistent with the metazoan data (120), CG methylation was found to be higher in WT exons compared with adjacent introns, and was not solely dependent on the CG composition of exons and introns (120). Further evidence from cotton showed similar CG methylation levels in constitutive and alternative exons, but variable patterns during different fiber development stages (121). By contrast, CG methylation was higher in alternative introns than constitutive introns. Furthermore, differential CG methylation has a strong influence on nucleosome formation since constitutive exons displayed higher nucleosome occupancy than alternative exons. However, alternative exons exhibited higher nucleosome density than constitutive introns (121). These findings clearly demonstrate that the relationship between DNA methylation and nucleosome occupancy is conserved between animals and plants, and AS is also predominantly regulated at the chromatin level in plants (42,82,92).

## HISTONE REMODELING MODULATES ALTERNATIVE SPLICING IN PLANTS

**Figure 1.**
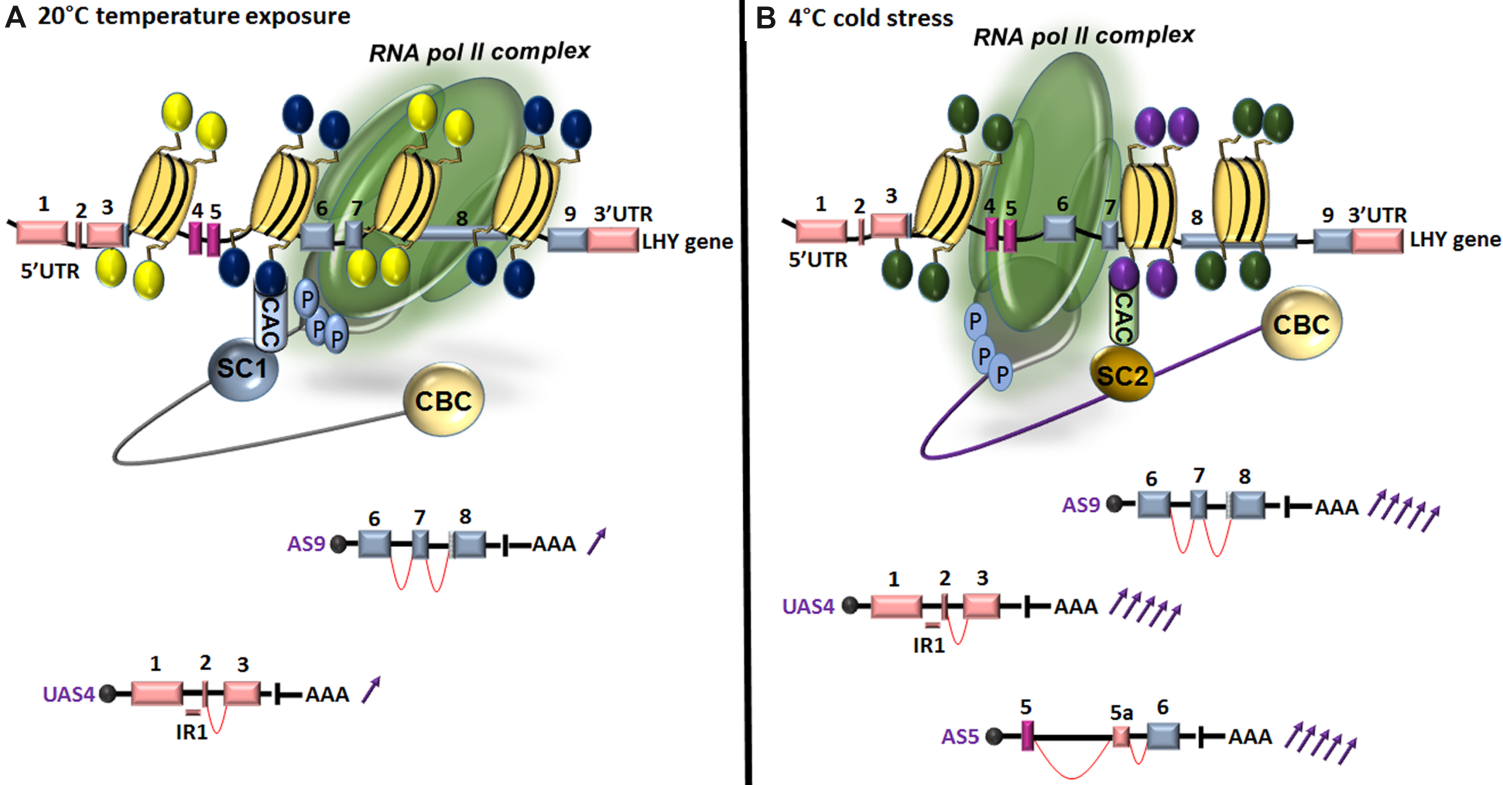
Schematic diagram illustrating proposed histone modifications and co-transcriptional splicing mechanisms in response to 22^o^C (A) and 4^o^C (B) using the *LHY* gene as an example. Temperature-dependent alternative splicing of the *LHY* gene generates different transcripts with variable abundance (purple arrows). For clarity, only a part of each splice variant is shown. At 4°C, both splice isoforms (UAS4 and AS9) are elevated from 10% (one arrow) to 50% (five arrows), and a new isoform (AS5) is produced (19). Under different temperatures, nucleosome (yellow disks) enrichment with single or combined histone marks (yellow, dark blue, green and purple circles) may mediate the RNA pol II (green oval) elongation rate and subsequently the differential recruitment of splicing factors complex (SC1/2) through readers and chromatin-adaptor complexes (CACs) to modulate cold-specific splicing. Light blue circles labeled ‘P’ and the gray teardrop represent phosphorylated CTD. UAS4 represents an intron retention (IR1) event in the 5′-untranslated region (UTR). AS9 removes three nucleotides via an Alt3′ in exon 8. AS5 adds an alternative exon 5a of 82 nucleotides via an alternative Alt3′ and Alt5′. Exons are displayed as numbered boxes, introns as lines. Myb-encoding exons are purple, exons in the 5′/3′-UTRs and coding sequence are shown in pink and light blue, respectively. Gray circles and AAA represent the 7-methylguanosine cap and poly-A tail, respectively. Red arcs represent the intervening sequence between 5′ss and 3′ss for different AS events.

Since transcription by pol II is affected by chromatin structure, it is unsurprising that stress-induced chromatin modifications can affect co-transcriptional splicing outcomes in plants. To fully understand the influence of chromatin changes on co-transcriptional AS, stress-induced DNA methylation and histone modification should be considered interconnected processes. Plant responses to environmental stress have been linked to modification of histone *N*-tails (34,122,123). However, it is important to understand whether transcriptional regulation mediated by histone modifications can also influence AS. Indeed, emerging evidence indicate the role of single or combined histone marks in AS regulation in plants (34,36). For example, PRMT5 methyltransferase (also known as SKB1) increases H4R3sme2 (histone 4 arginine 3 symmetric demethylation) levels in Arabidopsis to suppress the transcription of *FLC* and a number of stress-responsive genes (124,125). Upon salt stress, SKB1 disassociation from chromatin results in a reduction in the cellular levels of H4R3sme2, resulting in the induction of *FLC* and salt stress-responsive genes through higher methylation of the small nuclear ribonucleoprotein Sm-like4 (LSM4) (125). In addition, *skb1* mutants display pre-mRNA splicing defects caused by reduced symmetric dimethylation of arginine in LSM4 (125). These results demonstrate that SKB1 alters the methylation status of H4R3sme2 and LSM4 to link transcription and pre-mRNA splicing during stress responses. Additionally, PRMT5 also alters AS in the core clock gene *PSEUDO RESPONSE REGULATOR* 9 (*PRR9*) and influences clock functioning in Arabidopsis (126). Similarly, recent evidence in rice indicate that histone H3K36-specific methyltransferase (SDG725) regulates IR events in many genes (36). These IR events are much more prevalent at the 5′ end of gene bodies, and accompanied by high H3K36me2 histone marks, whereas the 3′ end of gene bodies are associated with fewer IR events and minimal H3K36me2 accumulation (36). Furthermore, IR shifts along the ends of gene bodies are more significant when both H3K36me2 and H3K36me3 modifications occur simultaneously (36). In Arabidopsis, temperature-induced differentially spliced genes are enriched in H3K36me3 marks to induce flowering (34). By contrast, depletion of H3k36me3 marks has the opposite effect to temperature-induced AS (34). It is possible that plants remember temperature variation via H3k36m3 and associated splicing patterns to influence flowering. Taken together, these studies indicate that stress-induced specific changes in histone PTMs may alter the chromatin landscape to mediate AS patterns in plants. A model illustrating how histone PTMs may regulate AS in response to temperature is presented in Figure 1.

## CHROMATIN-ADAPTOR COMPLEXES: KEY INTEGRATORS OF SPLICING FACTOR RECRUITMENT

Chromatin state not only affects pol II speed to modulate AS outcomes, but also promotes differential recruitment of SFs through chromatin adaptor complexes (75). The best example of a chromatin splicing adaptor complex in a mammalian system is AS of the human fibroblast growth factor receptor 2 (*FGFR2*) gene (75). H3/K36me3 recruits polypyrimidine tract binding protein (PTB) SFs to exon IIIb of *FGFR2* via the histone tail-binding protein MORF-related gene 15 (MRG15), suggesting that adaptor systems can regulate histone-dependent AS (75). Similarly, the role of adaptor complexes in regulating AS has also been reported in Arabidopsis (127). MORF-RELATED GENE 1 (MRG1) and MRG2 in Arabidopsis are homologs of human MRG15, and can bind H3K36m3-modified histones in a similar manner to MRG15. In plants, MRG1/2 proteins can trigger temperature-induced flowering via AS of flowering-related genes in WT plants. On the other hand, *mrg1-1 and mrg2*-*3* mutant plants lacking H3K36me3 readers display less sensitivity to temperature-induced flowering, implying a role for MRG adapters in regulating splicing variation and flowering (127). Similarly, the SMU2 protein was identified as an auxiliary factor of spliceosomal proteins in maize and Arabidopsis that modulates splicing of similar target pre-mRNAs in both species (128). SUM2 may facilitate the recruitment of chromatin modifier complexes to an alternative exon, thereby mediating AS of genes with specific chromatin features (128). Collectively, these reports highlight the importance of plant chromatin adaptor complexes in integrating condition-dependent histone modifications into a splicing code. This might explain how plants respond to stressful conditions through epigenetic regulation of AS (Figure 1).

## THE EPITRANSCRIPTOME: A REGULATOR OF SPLICING VARIATION

Chemical modification of RNAs, collectively referred to as the epitranscriptome, adds another layer of complexity to pre-mRNA splicing (129,130). In mammals and plants, m^6^A is the most abundant RNA modification, and is involved in the regulation of RNA processing (131–133). In mammals, co-transcriptional m^6^A deposition near splice sites promotes high splicing kinetics. However, high m^6^A levels in introns are associated with slow pol II processivity and AS of nascent RNA transcripts (132). M^6^A is also considered a post-transcriptional regulator of pre-mRNA splicing (134). In mammals, m^6^A recruits the mRNA methylation reader YTHDC, which in turn recruits SR proteins to their corresponding binding sites (134). Additionally, m^6^A facilitates recruitment of hnRNP C, a key player in pre-mRNA splicing, to regulate levels of alternatively spliced transcripts (134). In another study, the presence of TATA boxes was found to enhance the pol II elongation rate in humans (32). This decreases the time window for recruitment and physical attachment of RNA N6-adenosine-methyltransferase-like 3 (METTL3; an enzyme that methylates adenosine residues of some RNAs) to pol II CTD, lowering m^6^A modification of mRNAs (32). Interestingly, mRNAs with low m^6^A levels displayed increased translation efficiency, which was not the case for m^6^A-rich transcripts (32).

**Figure 2.**
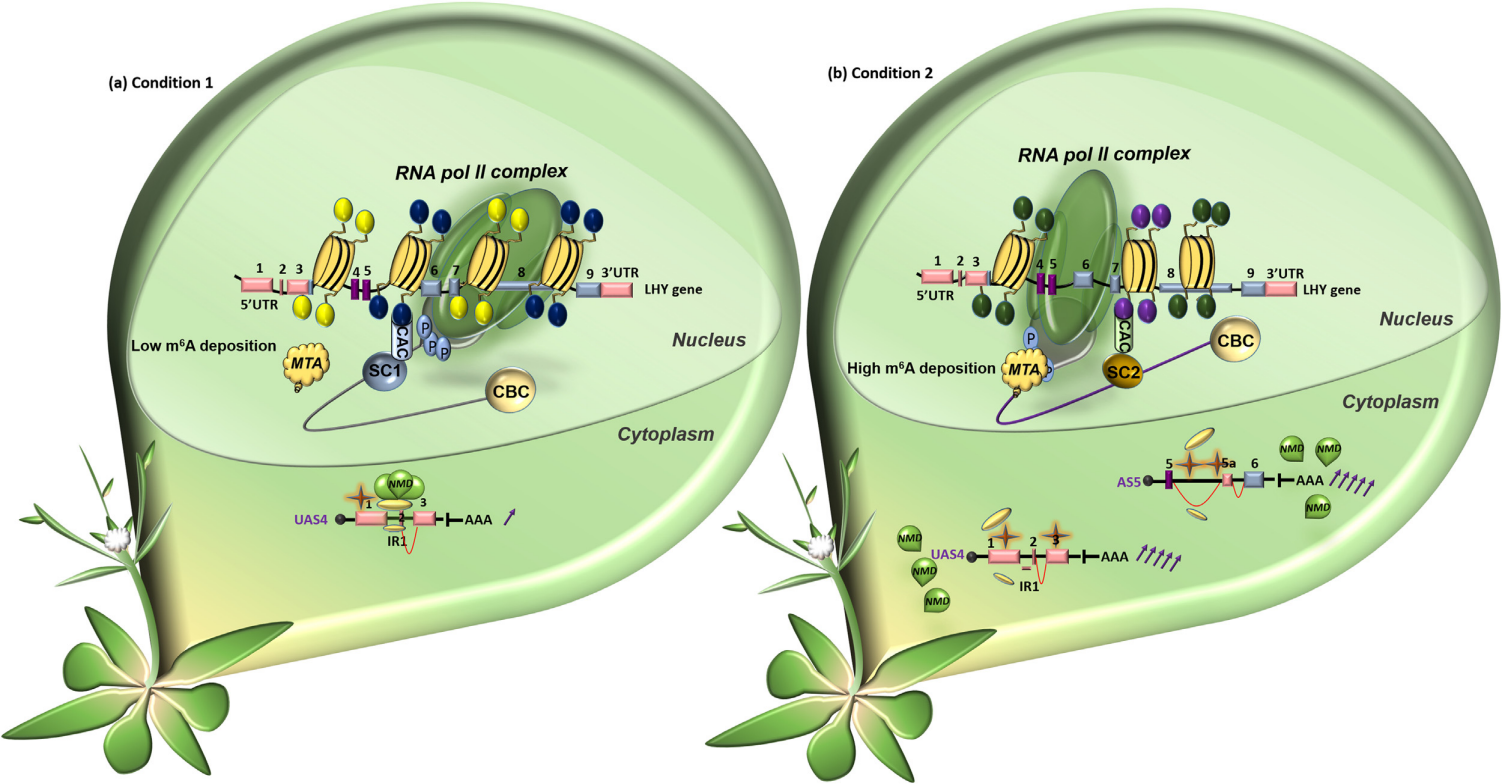
Model illustrating how condition-specific epigenetic marks may affect the rate of RNA pol II elongation, RNA base modification(s) and the fate of splice isoforms. Two NMD-sensitive splice isoforms of the *LHY* gene are used as hypothetical examples here. A fast RNA pol II elongation rate disables methyltransferase (MTA) recruitment, resulting in low m^6^A deposition (brown stars) over UAS4 (**A**). Slow RNA pol II elongation enables MTA recruitment and mediates high m^6^A deposition over UAS4 and AS5 (**B**). Low m^6^A deposition allows efficient ribosome (gold spheres) loading and facilitates NMD recruitment (A), whereas the opposite is true for USA4 and AS5 in condition (B). Hence, condition-specific histone modifications (shown as yellow, dark blue, green and purple circles) and differential nucleosome occupancy (yellow disks) may regulate the RNA pol II elongation rate to assist NMD-sensitive transcripts (UAS4 and AS5) escape degradation. *LHY* splice variants UAS4 and AS5 display sensitivity to NMD only under certain conditions (19). The abundance of each transcript under different conditions and relative to each transcript within the same condition is denoted with purple arrows. For labels explanation, see Figure 1 legend.

In Arabidopsis, high-throughput annotation of modified ribonucleotides (HAMR) revealed that chemical modification of RNA differentially marks the vicinity around splice donor/acceptor sites of alternatively spliced introns within stable mRNAs (i.e. 3-methylcytidine) (135). Recent global run-on sequencing (5′GRO-seq) data from Arabidopsis showed that most gene promoters are strongly enriched in AT nucleotides, implying a role for TATA box-mediated transcription (136). Although transcriptional regulation at the level of initiation is beneficial for plants by facilitating rapid responses under variable environmental conditions, additional control via RNA modification may be employed to dynamically control the fate of a given transcript. Therefore, it is tempting to speculate that co-transcriptional RNA modifications (m^6^A or other marks), which are highly prevalent in plant mRNAs (137,138), may play a role in regulating splicing outcomes and the translational fate of different transcripts in plants (Figure 2). However, more robust methods and tissue/condition-specific profiling are needed to illuminate the mechanisms by which epitranscriptomic changes regulate splicing and the translational outcomes of fully spliced and AS transcripts.

## ENGINEERING SPLICING VARIATION

RNA interference (RNAi) has been the gold standard for silencing targeted genes (139,140). However, the advent of CRISPR/Cas9-driven strategies has revolutionized the way we are able to modulate the expression (and possibly splicing) of single or multiple genes at the DNA level with greater target specificity (141). Recently, an RNA-guided RNA targeting CRISPR/Cas13 system has been developed for transcriptional regulation (142). Development of RNA-specific technologies such as Cas13 has increased the power with which we now can silence virtually any gene with a corresponding matching guide CRISPR RNA (crRNA) that guides the Cas13 protein to its target RNA (143). In addition, the development of tissue-specific pol II-driven promoter systems, coupled with self-cleaving ribozyme and tRNAs flanking the desired guide RNAs (gRNAs), has made it possible to express gRNAs from any desirable promoter, providing unprecedented cell and tissue specificity (144–146). Development of Cas9 and Cas13 systems to modulate transcriptional and post-transcriptional outcomes opens up exciting new possibilities for engineering transcriptomes (147). Modulating gene expression patterns in a given generation or at a specific time point is important. However, the ultimate challenge is to develop CRISPR arrays that can modulate the expression and splicing of many genes through multiple generations. Stable inheritance of differentially methylated regions has been demonstrated to mediate extensive phenotypic variation in many traits in plants, and to contribute to observable heritable traits, which is explained by epi-alleles (148). It is now possible to modulate methylation of target loci using CRISPR/deadCas9 systems coupled with methylation/demethylation enzymes to engineer important traits such as flowering (149). Since DNA methylation and histone modifications modulate splicing outcomes in concert with pol II speed in many species (23,116,120,150), designing splicing and isoform expression patterns in a tissue- and growth-specific manner has become feasible. For example, the *FLOWERING LOCUS M* (*FLM*) gene exhibits temperature-dependent AS and regulates flowering in Arabidopsis (14). Recently, CRISPR/Cas9 technology was used to probe the roles of the two splice variants of *FLM* (*FLM-β* and *FLM-δ*) by deleting exons 2 and 3, respectively (151). Lines producing repressive FLM-β but not FLM-δ flowered late, whereas lines producing *FLM-δ* displayed early flowering, suggesting that splice variant β acts as a flowering suppressor (151).

Since translation and ribosomal loading of transcripts are mediated by the circadian clock and photoperiodic length in plants (152,153), the timing of expression should also be taken into consideration when designing CRISPR arrays, since coincidence with natural or WT expression contexts could reap maximum benefits. Even if translation of a particular protein is desired at a time different from that occurring naturally, Cas13 systems coupled with RNA methylation readers, writers or erasers could be combined to carve desirable methylation patterns and thereby enhance or suppress translation (32). We envisage that further refinement of CRISPR/Cas strategies and the availability of versatile vectors and arrays will facilitate targeting of multiple genes for different outcomes simultaneously (144–146,154,155). Although CRISPR systems have revolutionized the way we edit genomes on a global basis, we believe that chromatin context, which may provide a timing and regulatory framework, will remain relevant; hence, we must understand the chromatin language (156) before engineering biological networks at will.

## CONCLUDING REMARKS

**Figure 3.**
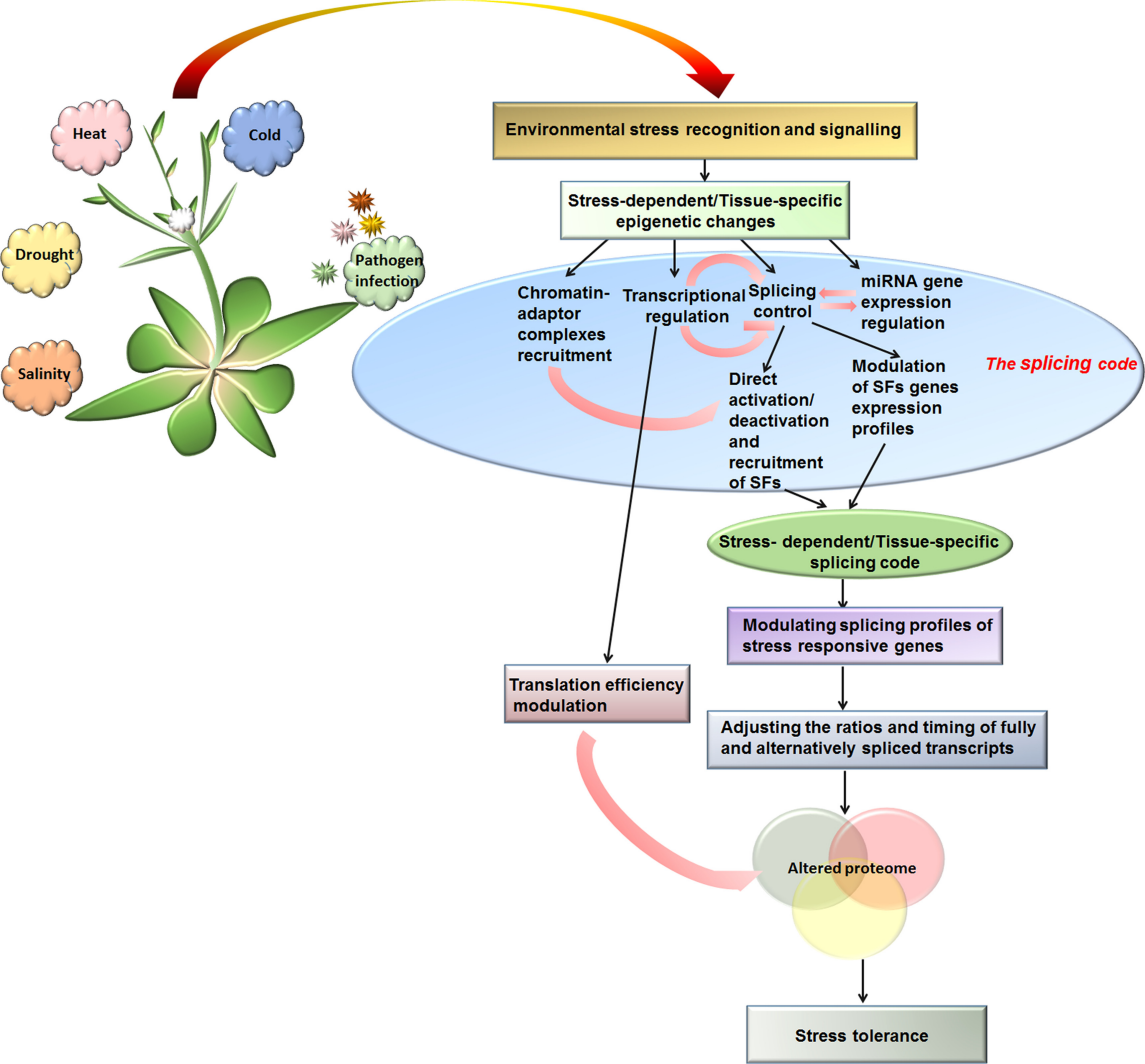
Schematic diagram showing how the stress-induced splicing code may promote stress tolerance. Variable environmental conditions alter chromatin structure, regulating transcriptional and splicing dynamics and modulating the expression of stress-responsive genes. Stress-induced epigenetic modifications result in a condition-specific splicing code through the differential recruitment of chromatin-adaptor complexes and/or micro RNA (miRNA) regulation. The stress-specific splicing code can fine-tune the expression of target genes by adjusting transcript ratios and timing, triggering appropriate changes in transcriptome and proteome composition, thereby conferring adaptive responses under stress conditions.

A growing body of evidence acquired in recent years suggest that co-transcriptional splicing regulation mediated by epigenetic mechanisms occurs in both animals and plants. In particular, pol II initiation and elongation speed mediate the co-transcriptional processing of pre-mRNAs, and modulate the abundance of constitutive and AS transcripts in animals and plants. In plants, DNA methylation and epigenetic modifications regulate splicing patterns of pre-mRNAs of some genes. Although a direct link between epigenetic modifications and AS in plants is yet to be established, emerging epigenetic engineering approaches should address this in the future. Further work is needed to illuminate the complex regulatory mechanisms controlling splice isoform ratios in a cell-type and condition-specific manner (Figure 3). The next steps are to determine how the splicing code is ‘built’ from epigenetic and epitranscriptomic modifications, and reveal how it can modulate (i) the timing required to process different pre-mRNAs in an pol II speed-dependent manner and (ii) the ratios of fully and alternatively spliced transcripts to produce the desirable transcriptome under different conditions. To help answer these and other questions, we must determine the translation efficiency of alternatively spliced transcripts, and reveal how plants fine-tune their proteome at co/post-transcriptional levels, as well as translational/post-translational levels, by directing their transcripts to NMD or nuclear retention. It would also be useful to investigate how RNA methylation patterns are established and preserved after pre-mRNA synthesis and maturation into mRNAs in plants. Addressing these questions will undoubtedly expand our understanding of the chromatin code in plants.
